# Metagenomic nanopore sequencing of ocular microbiome in patients with meibomian gland dysfunction

**DOI:** 10.3389/fmed.2022.1045990

**Published:** 2022-11-09

**Authors:** Dalan Jing, Xiaodan Jiang, Xiaotong Ren, Jie Su, Chen Huang, Jiarui Yang, Ran Hao, Xuemin Li

**Affiliations:** ^1^Department of Ophthalmology, Peking University Third Hospital, Beijing, China; ^2^Beijing Key Laboratory of Restoration of Damaged Ocular Nerve, Peking University Third Hospital, Beijing, China; ^3^Medical Research Center, Peking University Third Hospital, Beijing, China; ^4^Beijing Ophthalmology and Visual Sciences Key Laboratory, Beijing Tongren Eye Center, Beijing Tongren Hospital, Capital Medical University, Beijing, China

**Keywords:** metagenomic nanopore sequencing, meibomian gland dysfunction, microbiome, bacterial flora, microbiota imbalance

## Abstract

**Purpose:**

To explore the composition of the ocular microbiome in patients with Meibomian gland dysfunction (MGD) using metagenomic nanopore sequencing.

**Methods:**

A total of 98 participants were recruited from September to December 2021, including 86 patients with MGD and 12 controls. Symptoms and signs of dry eye were assessed, and bacterial samples in the conjunctival sac (CS) and meibomian gland (MG) secretions were then identified by bacterial culture identification and metagenomic nanopore sequencing.

**Results:**

The positive rate of CS bacterial culture in the MGD group was significantly higher than that in the normal group. A more complex composition of bacterial genera was detected in the mild and moderate MGD groups than in the control. However, the severe MGD groups had the simplest composition of bacteria. Metagenomic nanopore sequencing detected more species of bacteria than traditional culture.

**Conclusion:**

The CS and MG of MGD patients may have different degrees of bacterial microbiota imbalance. Metagenomic nanopore sequencing technology provides a new way for us to understand the composition of “real-world” ocular surface microorganisms.

## Introduction

Symbiotic bacteria on the ocular surface have been controversial because of tear washing and ocular antibacterial substance secretion. Recent studies have demonstrated the colonization of ocular surface flora, including a variety of bacteria, fungi, viruses, etc. (1, 2). Healthy eye microbiota is a low-diversity microbiome, in which the Staphylococcus was most common (3). Changes in microbiome ecology significantly affect ocular homeostasis, and shifts can also affect the progression of ocular surface diseases (4). The changes in microbiota in eyes are related to several disease states, including blepharitis, conjunctivitis, keratitis, and dry eye syndrome (5–8).

Meibomian gland dysfunction (MGD) is a multifactorial chronic eyelid disease that causes irritation, inflammation, and ocular surface disease. There is some evidence that patients with MGD have an overgrowth of ocular flora, and the severity of MGD appeared to be positively correlated with a higher bacterial complexity (9). Topical antibiotics, such as azithromycin alone or in combination with doxycycline, can improve MGD symptoms both by altering the ocular flora and through anti-inflammatory mechanisms (10). It is important to explore the ocular bacterial composition. Various techniques can be used for ocular surface microbial detection. The composition of the ocular microbiota may vary according to the analytical method. A previous study using traditional cultivation inevitably neglected some bacteria that had low abundance or had to be cultured (4), but traditional cultivation remain the gold standard for diagnosis of ocular infections. Gene analysis with the universal primers in 16S rDNA using next generation sequencer (NGS) has been applied in the analysis of ocular infectious diseases (11, 12). Dong et al. (13) found that patients with MGD can have various degrees of bacterial microbiota imbalance in the conjunctival sac (CS) using 16S rDNA gene sequencing. Due to the limitation of Illumina sequencing platform—short-read (500 base pairs), only part of the 16SrDNA gene can be sequenced, thus limiting the taxonomic resolution to genus-level classification (14). Metagenomic NGS enabled to provide all the components of the microbiome (15). Doan and Pinsky (16) showed how the healthy ocular surface has a unique microbiome with viral and bacterial communities. Parekh et al. (17) confirmed the diagnosis of herpes simplex virus infection of cornea along with the taxonomical profiling of the virus. But Illumina sequencing platform has high operating costs, which means that these facilities can only be used in specific centers.

MinION, a long- read portable nanopore sequencer, overcomes the disadvantages of NGS and has been used in bacterial ocular infections which can rapidly and accurately identifying infection bacteria including corneal ulcers, endophthalmitis, and conjunctival abscess (18). Besides, it enables rapid library preparation and real-time analysis, which helps reduce the turnaround time. Metagenomic nanopore sequencing provides an agnostic method for detecting emerging pathogens directly from clinical specimens. Compared with targeted methods, it also provides valuable information about the composition of the microbiome and might uncover coinfections that may be related to disease progression and impact prognosis (19).

The purpose of this study was to investigate the normal and MGD ocular surface bacterial flora. Bacterial identification in conjunctival swab and MG secretions was assessed by metagenomic nanopore sequencing and conventional culture.

## Materials and methods

### Participants

Our study adhered to the tenets of the Declaration of Helsinki and was approved by the Ethics Committee of the Peking University Third Hospital (S2021222). We obtained written informed consent from all patients before the examination.

A total of 98 participants were recruited from September to December 2021, including 86 patients with MGD (36 males and 50 females, 29.24 ± 6.15 years old) and 12 controls without MGD (5 males and 7 females, 27.36 ± 3.25 years old). The diagnosis of MGD is based on clinical signs and symptoms and ocular examinations, including slit-lamp microscopy, tear break-up time (TBUT), tear meniscus height (TMH), and meibography. Participants were eligible if the patients were diagnosed with MGD based on the 2011 International Workshop on MGD (20). MGD patients were divided into groups of mild MGD, moderate MGD and severe MGD depending on eyelid margin signs, clinical symptoms, and quality of meibum (21). The inclusion criteria for the controls were MG related assessment failed to meet MGD diagnostic criteria and no chief complaint of any dry eye symptoms. Patients with contact lens wear within 1 month, eye drops, or oral antibiotics use within 2 weeks, recent eye surgery, nasolacrimal duct obstruction, corneal diseases, eye trauma, eye infection, glaucoma, and ocular fundus diseases were excluded from the study population.

### Clinical evaluation

The clinical assessments of the enrolled participants were conducted in the following order at the clinical first visit: collection of demographic information, working hours on a computer, sleeping time, visual analog scales of 10 specific symptoms that were used to assess the patient’s subjective symptoms (dryness, foreign body sensation, ache, burning, tearing, asthenopia, blur, itching, secretions, and photophobia) (22), the Ocular Surface Disease Index (OSDI) questionnaire (23), slit-lamp biomicroscopy including conjunctival injection (24), eyelid margin and MG assessments (20). The keratograph 5 M (Oculus Optikgeräte GmbH, Wetzlar, Germany) was used to measure the non-invasive TBUT, TMH and meibography scores (22, 23).

### Sample collection

Samples were collected in a sterile treatment room by a sample ophthalmologist (JDL) who wore sterile gloves and masks. Then, a drop of 0.4% Oxybuprocaine Hydrochloride (Benoxi; Unimed Pharma Ltd., Slovakia) was applied to eye as topical anesthesia. The eyelid margins (including the roots of the eyelashes) were sterilized using entoiodine. Sterile swabs were used to collect bacterial samples from the lower CS and MG secretions. The operation needs to be repeated twice. In addition, sterile swabs were exposed to the air of the operating room for 10 s each time as a blank control. All the samples were placed in sterile Eppendorf tubes (Axygen, USA) and stored at 4°C.

### Conventional bacterial culture

Samples were incubated on blood agar and chocolate agar plates at 35°C for 48 h. Bacterial identification was performed from the positive culture plate using standard biochemical tests by a VITEK 2 Compact system (bioMe’rieux, France).

### Metagenomic nanopore sequencing

DNA was extracted from the samples using the Quick-DNA/RNA Viral Kit (Zymo Research, USA). Barcode sequencing libraries were generated using the Rapid Barcoding Sequencing kit (SQK-RBK004) following the manufacturer’s protocol (Oxford Nanopore Technologies, Oxford, UK). Then, nanopore sequencing was performed on a MinION system (Oxford Nanopore Technologies, Oxford, UK). After sequencing, the generated reads were analyzed with EPI2ME (Oxford Nanopore Technologies, Oxford, UK). FASTQ WIMP (What’s in My Pot), which is a cloud-based data analysis platform. For nanopore sequencing, unique reads > 3 for bacteria and unique reads > 1 for fungi were considered positive for pathogenic microorganism identification (25).

### Statistical analysis

All analyses were performed using SPSS version 23.0 software (Chicago, IL, USA). ANOVA was used to compare the differences in age, sex, and clinical exam results between the patients with MGD and the controls. To compare the positive rate of bacterial culture of CS and MG secretion, chi square test was used. To compare the overall analysis of the positive rate of different bacteria cultured on the ocular surface between different groups, chi square test was used. Statistical significance was set at *p* < 0.05.

## Results

### Patient demographic and ocular characteristics

A total of 98 subjects were recruited (86 MGD eyes and 12 control eyes). Age and sex were matched between the MGD and control groups (*P* = 0.25 and *P* = 0.59, respectively). Of these 86 eyes with MGD, 49 were subcategorized into the mild MGD group, 28 into the moderate MGD group and 9 into the severe MGD group. All patients completed the information collection process and examinations, and the results are shown in Table 1.

**TABLE 1:** Basic characteristics of MGD patients and controls.

Objectives	Control (*n* = 12)	Mild (*n* = 49)	Moderate (*n* = 28)	Severe (*n* = 9)	*P*
Age (years)	27.36 ± 3.25	28.21 ± 4.87	29.14 ± 5.72	28.42 ± 6.64	0.17
Sex, male (%)	5 (41.7%)	26 (53.1%)	6 (21.4%)	4 (44.4%)	0.43
Computer time (h)	8.93 ± 2.16	8.09 ± 2.80	8.54 ± 3.10	8.71 ± 1.98	0.72
Sleeping time	1.71 ± 0.47	2.02 ± 0.26	1.96 ± 0.19	2.14 ± 0.36	0.02*
OSDI score	13.54 ± 5.88	29.36 ± 7.08	31.91 ± 6.12	47.38 ± 15.73	0.04*
Total sign score	18.21 ± 7.00	24.48 ± 3.99	25.32 ± 11.03	40.35 ± 10.24	0.02*
TBUT	12.43 ± 3.56	6.02 ± 1.29	4.28 ± 1.27	2.14 ± 2.54	0.50
TMH	0.18 ± 0.02	0.18 ± 0.06	0.18 ± 0.05	0.18 ± 0.04	0.90
Eye margin score	0.50 ± 0.52	1.97 ± 1.13	3.89 ± 0.80	5.43 ± 0.65	< 0.01*
MG exclusion score	0.58 ± 0.45	1.40 ± 0.62	2.32 ± 0.61	2.69 ± 0.31	< 0.01*
Meibum score	0.42 ± 0.50	0.86 ± 0.45	1.37 ± 0.65	1.94 ± 0.81	< 0.01*
Meiboscore	0.57 ± 0.25	1.40 ± 0.62	2.32 ± 0.61	2.69 ± 0.30	< 0.01*
MG score	3.36 ± 1.39	6.02 ± 1.29	7.59 ± 3.27	9.64 ± 1.90	< 0.01*

*n*, number of eyes; MGD, meibomian gland dysfunction; OSDI, ocular surface disease index; TBUT, tear break-up time; TMH, tear meniscus height; MG, meibomian gland.

*P < 0.05.

### Biochemical identification results of bacterial culture

The microbiomes isolated from CS and MG secretions are shown in Table 2. *S. aureus* was the most common bacteria isolated from CS, while *S. epidermidis* was the most common bacteria isolated from MG. In both the CS and MG, most of the isolated bacteria were gram-positive. However, Gram-negative bacteria can be isolated from MG, including *C. koseri*, which is a conditional pathogenic bacterium. No bacteria were isolated from CS (96.9%) or MG (80.6%) in most patients. The bacterial culture positive rate of MG was significantly higher than that of CS (*P* < 0.01). In controls, culture positivity of CS was 0%; however, culture positivity of MG secretion was 16.7%. In the MG, the positive rate of CS bacterial culture was 4.7%, while the positive rate of bacterial culture of MG secretion was 20.9%. The positive rate of CS bacterial culture in the MGD group was significantly higher than that in the normal group (*P* = 0.04). However, there was no significant difference in the positive rate of MG bacterial culture between the two groups (*P* = 0.53).

**TABLE 2:** Results of bacterial culture of conjunctival sac (CS) and meibomian gland (MG).

Isolated bacteria	*n* (%)	*n* (%)	*P*
	CS	MG	
None	95 (96.9%)	79 (80.6%)	<0.01*
***Staphylococcus* (G +)**			
*S. epidermidis*	1 (1.0%)	17 (17.3%)	<0.01*
*S. aureus*	2 (2.0%)	3 (3.11%)	0.65
*S. auratus*	1 (1.0%)	0	0.32
*S. hominis*	1 (1.0%)	0	0.32
***Citrobacter* (G-)**			
*C. koseri*	0	1 (1.0%)	0.32

*n*, number of eyes; G +,gram positive bacteria; G–, gram negative bacteria.

*P < 0.05.

### Metagenomic nanopore sequencing partial results

Table 3 summarizes the partial results of the nanopore sequencing. The results of the two identification methods are slightly different for the same sample. Twenty-five positive samples of bacterial culture were tested. The nanopore sequencing reading length was longer, and more information was compared to the database, which improved the identification of bacteria at the species level. From the results, the number of sequences aligned to the species level accounted for 77.7–95.2% of the genus level. The N50 reading length of the sequencing data of all samples was 2.4–3.2 kb, the reading length was 1,378–2,085 bp, and the *Q*-value of the mass fraction was 11.02–12.74. In addition to detecting bacteria on the ocular surface, nanopore technology also found the presence of a significant amount of virus in MG secretions for the first time, including *Phietavirus*, *Biseptimavirus* and *Triavirus*, which belong to *Staphylococcus* virus.^1^

**TABLE 3:** Nanopore sequencing partial results.

Isolated bacteria	*N* (%)	*N* (%)	*P*
	CS	MG	
None	95 (96.9%)	79 (80.6%)	< 0.01 *
***Staphylococcus* (G +)**			
*S. epidermidis*	4 (4.1%)	19 (19.4%)	< 0.01 *
*S. saprophyticus*	1 (1.0%)	14 (14.3%)	< 0.01 *
*S. lugdunensis*	1 (1.0%)	14 (14.3%)	<0.01
*S. capitis*	4 (4.1%)	19 (19.4%)	< 0.01 *
*S. heamolyticus*	2 (2.0%)	17 (17.3%)	< 0.01 *
*S. warneri*	1 (1.0%)	6 (6.1%)	0.12
*S. argenteus*	1 (1.0%)	5 (5.1%)	0.13
*S. equorum*	0	7 (7.1%)	0.02 *
*S. pseudintermedius*	0	3 (3.1%)	0.25
*S. simulans*	1 (1.0%)	8 (8.2%)	0.04 *
*S. pasteuri*	2 (2.0%)	10 (10.2%)	0.04 *
*S. nepalensis*	0	3 (3.1%)	0.25
*S. simiae*	0	2 (2.0%)	0.48
*S. condimenti*	0	3 (3.1%)	0.25
*S. xylosus*	1 (1.0%)	0	0.32
*S. carnosus*	0	1 (1.0%)	0.32
*S. schleiferi*	0	1 (1.0%)	0.32

*n*, number of eyes; CS, conjunctival sac; MG, meibomian gland; G +,gram positive bacteria; G–, gram negative bacteria.

*P < 0.05.

### Relationship between two identification results

We calculated the matching rate according to whether the dominant flora of the two was consistent and then categorized them as perfect match, incomplete match and complete mismatch (23/24; 0/24; 1/24). Biochemical analysis flora species: A total of 1 genus and 4 species of bacteria were isolated from CS; two genera and three species of bacteria were isolated from MG; and a total of 2 genera and 5 species of bacteria were isolated. Nanopore analysis of bacterial species: a total of 7 genera and 18 species of bacteria were isolated from CS; a total of 14 genera and 40 species of bacteria were isolated from the MG; and a total of 18 genera and 44 species of bacteria were isolated.

### Compositions of the microbiomes of the meibomian gland dysfunction groups and healthy controls

**FIGURE 1:**
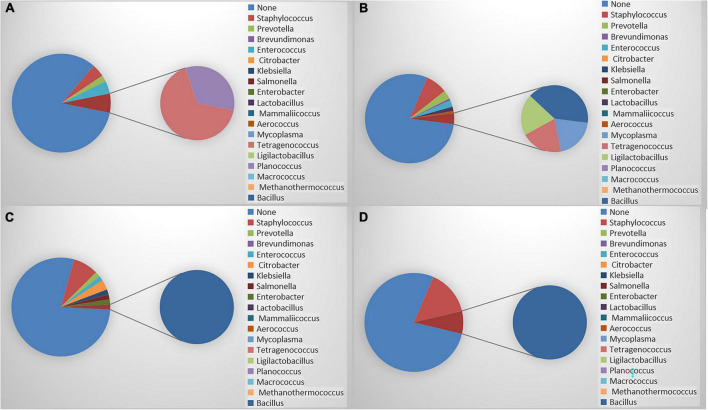
**(A)** Composition of meibomian gland bacteria in control group; **(B)** composition of meibomian gland bacteria in mild MGD; **(C)** composition of meibomian gland bacteria in moderate MGD; **(D)** composition of meibomian gland bacteria in severe MGD.

As shown in Figure 1, a more complex composition of bacterial genera was detected in the mild and moderate MGD groups than in the control. Interestingly, the severe MGD groups had the simplest composition of bacteria. *S. epidermidis* is the most isolated bacterium on the ocular surface (26). Isolation rates were analyzed with or without the inclusion of *S. epidermidis*. The positive isolation rate of *S. epidermidis* in the severe MGD group was significantly lower than that in the other groups. However, the mild MGD group had the highest positive isolation rate of *S. epidermidis*. When we analyzed the data without S. *epidermidis*, the severe MGD group had the highest positive isolation rate of other bacteria, but the control and mild MGD group were significantly lower than that in the other groups (Table 4).

**TABLE 4:** Characteristics of isolated bacterial flora in control and different MGD severity.

Objectives	Control (*n* = 12)	Mild (*n* = 49)	Moderate (*n* = 28)	Severe (*n* = 9)	*p*
With s.e. (*n*)	2	10	5	0	<0.01*
Without s.e. (*n*)	0	0	1	3	0.04*

MGD, meibomian gland dysfunction; s.e., *staphylococcus epidermidis; n*, number of eyes.

*P < 0.05.

## Discussion

MG itself is adjacent to the eyelid skin and conjunctiva, which feet with bacteria. Speaker et al. confirmed that the pathogens cultured in the vitreous of 82% of endophthalmitis patients were the same as those in the eyelid, conjunctiva, or nasal cavity of the patients (27). Karp et al. considered that the infection of *S. aureus* and coagulase-negative *Staphylococcus* after LASIK is most likely related to blepharitis and MGD (28). Subjects with significant MGD or blepharitis should be instructed to perform lid hygiene or oral doxycycline before operation. Our previous study showed that the MG was secreted during the operation even if antibiotics, CS irrigation and povidone iodine were used before the operation (29). The preoperative treatment of MGD, the application of intraoperative surgical film, and the application of postoperative antibacterial eye drops are very important.

The deposition of bacteria-associated molecular substances to the ocular surface may occur via the inflammation of MG orifices, thus resulting in ocular surface irritation related to MGD (30). Meanwhile, chronic, and long-term eyelid inflammation can damage ocular surface epithelial cells, lead to changes in the ocular surface microenvironment, reduce local resistance, and increase the risk of infection.

The microbial flora of the ocular surface has commonly been determined through conventional culture techniques, followed by the application of biochemical tests and morphological criteria to correctly identify the microorganism. However, traditional culture-based microbial studies have isolated a low diversity of microorganisms from the ocular surface (31). This methodology, as used in the present study, detected an overall higher level of conjunctival bacteria in patients with MGD than in normal subjects. This result is consistent with Zhang’s research, which showed that the CS in patients with MGD had more complex bacterial species than healthy individuals (4). There was no significant difference in the positive rate of MG bacterial culture between the two groups, which may signify changes in ocular surface flora in patients with MGD focusing on changes in bacterial composition.

The positive bacterial isolation rate for MG secretions was 19.4%, which was slightly lower than that in previous studies (4). Inconsistency among studies may be due to the different sample sizes, ages, races, sources of sampling and other methodological factors. Studies have shown that the microbial community composition changes with age. The species richness of the elderly group was significantly higher than that of the young group (32). Age appears to be a stronger factor in reshaping the ocular surface microbiome. The population included in our study is young people, which leads to the difference in the positive rate of bacterial culture. More research is needed to clarify the origin of this discrepancy. Conventional culture-based techniques rely on the phenotypic characteristics of microorganisms to estimate microbial load (33). Although it provides a rough assessment of microbial density and diversity in samples, these measurements are often inaccurate and biased. Another advantage is that it can identify live bacteria. The cultured microorganisms may only represent a portion that are easy to grow under standard culture conditions and may be only a small part of the real world (34). Moreover, some microorganisms cannot even be cultured on traditional laboratory media. Therefore, there are differences in the type and density of microorganisms that can be cultured from the eye surface in previous studies (6, 34). More advanced non-culture diagnostic methods are needed, such as metagenomic sequencing, which targets microbial RNA or DNA.

Compared with conventional culture-based studies, culture-independent-based studies have shown that the ocular surface has a richer microbial diversity. Dong et al. observed 24 genera, including pathogenic and non-pathogenic bacteria, on the healthy ocular surface by using 16S rDNA gene sequencing (1). A multicenter study using 16S rRNA sequencing method found an average of 88 genera on healthy ocular surface, and *Staphylococcus* was the most abundant genera (3). One study showed that the most prevalent microbes on the ocular surface were similar between patients with MGD and controls via 16S rDNA sequencing, but the bacterial relative abundance changed (13). The use of molecular techniques differs from cultivation. On the one hand, it improves our understanding of ocular surface biodiversity; on the other hand, because of its high sensitivity, it can monitor all existing microorganisms, whether they are alive or not, so there may be problems for clinical diagnosis.

Metagenomic nanopore sequencing could identify bacterial pathogens in MGD with a higher degree of sensitivity and faster processing time than conventional culture studies. In addition, greater bacterial diversity was detected at the genus and species levels in parallel samples. This method identified several additional common ocular bacteria, including *Streptococcus*. Some fewer common bacteria on the normal ocular surface, such as *Enterococcus*, *Klebsiella, Bacillus, Prevotella*, *Auricoccucs*, *Planococcus*, and *Tetragenococcus*, some may associate with infective conjunctivitis, keratitis and endophthalmitis. This finding suggested a potential association between ocular surface disorders and bacterial overgrowth or imbalance. *Enterococcus faecalis* was detected in 14 MG secretion samples by nanopore technology. routine culture temperature may be too high for it to grow, which reduces the likelihood of evaluating its pathogenic impact. In addition to detecting bacteria, a further limitation of metagenomic sequencing is the inability to assess the functional status of microbiota.

Increased bacterial diversity is considered beneficial because more diverse communities are usually more resistant to interference (35). However, in the case of eye diseases, including dry diseases, the diversity of eye microorganisms increases, possibly due to antimicrobial compounds in the tear film (36). When the number of bacteria reaches a high concentration, the increase in the number of bacteria may have a detrimental effect on normal cell function through the quorum sensing (QS) mechanism (37, 38). QS allows groups of bacteria to synchronously alter behavior in response to changes in the population density and species composition of the vicinal community. It is a complex chemical network behavior of several bacterial species for increasing their local population (39). In general, Gram-negative bacteria use acylated homoserine lactones as autoinducers, and Gram-positive bacteria use processed oligo-peptides to communicate (40). After reaching a certain concentration, these bacteria, including *S. aureus* and *P. aeruginosa*, have been shown to be able to initiate their toxicity and overcome the host immune response. *S. aureus* is one of the most common species isolated from the eyelid margin and meibum. The degree of pathogenicity exhibited by *S. aureus* depends on its successful invasion inside the tissue, and this process is a QS-associated phenomenon (41). At low cell density, the bacteria express proteins required for attachment and colonization, and as the cell density becomes higher, this expression profile switches to express proteins involved in toxin and protease secretion (37). *P. aeruginosa* has high resistance to antibiotics, ubiquitous nature, and potentially devastating effect on vision if infection arises (42). Complex QS system consisted of three circuits helps *P. aeruginosa* to survive at different environmental conditions, as well as to escape from host immune system (39). This may explain why the positive rate of ocular surface bacteria monitoring is increased in MGD patients. Although bacterial-related cytotoxicity or inflammation may contribute to the occurrence and progression of MGD, it is not surprising that patients often improve through antimicrobial treatments. Further studies are needed to explore the relationship between QS and bacterial expression of toxic factors in subjects with MGD, ocular inflammation, and elevated bacterial levels.

It is interesting that patients with severe MGD have reduced diversity of ocular surface flora and simple composition compared with mild and moderate. MGD is divided into high secretory MGD, low secretory MGD and obstructive MGD, of which obstructive MGD is considered the most common form (43). Overexpression of protein and underexpression of the lipid fraction reflect the viscosity of the expressed mebium leading to gland obstruction. The stagnation of MG secretion may promote the proliferation of eyelid bacteria inherent in the eyelid (44). In addition, blockage of gland pores may cause a hypoxic environment and promote the growth of anaerobic bacteria. Therefore, in mild to moderate MGD, bacterial types increased. These changes may increase the release of esterases and lipases by commensal bacteria of the eyelid. Due to this increase in enzyme activity, bacteria can change the viscosity of the meibomian plate, leading to further stagnation of the meibomian plate within the MG and production of free fatty acids, leading to inflammation and hyperkeratosis (45, 46). MG duct obstruction, hyperkeratosis and acinar cell dysfunction ultimately lead to MG degeneration. Toxins or proteases released by bacteria may damage glandular epithelial cells, leading to glandular atrophy in severe MGD (4). We can speculate that atrophy of the MG is accompanied by a reduction in secretion, the substrate on which bacteria grow is reduced, and only some bacteria with strong adaptability survive. This may also explain our finding that patients with severe MGD have reduced ocular surface flora types. In the analysis that did not include *S. epidermidis*, positive bacterial isolation rates were significantly higher in the severe MGD group than others which can also verify the changes of symbiotic bacteria on the ocular surface of severe MGD group from the side. Non-S. *epidermidis* may be related to the severity of the disease.

Moreover, *Phietavirus*, *Biseptimavirus* and *Triavirus* were found in MG secretions of MGD patients. *Phietavirus* is a common phage genus in *S. epidermidis* and *S. aureus*. *Phietaviruses* have transduction potential and a putative impact on the evolution of *S. epidermidis*. The decisive role of *S. epidermidis* phages in attaining a higher pathogenic potential of host strains. The study of these phages can promote our understanding of the changes in drug resistance of ocular surface bacteria in MGD patients. The genes of these phages need further study (47).

The results suggested that the ocular surface flora of MGD patients was changed. However, it is still unclear whether the change in the ocular surface microbiota is a cause or a consequence of the MGD and the microbial composition of healthy and MGD eye surfaces. More participants from a larger area and samples are needed for further investigations.

Metagenomic nanopore sequencing is a promising tool that could provide clinically relevant information including the presence or absence of resistance genes, from which the phenotype of resistance can be inferred (48). Nanopore sequencing has also been used to identify clinically relevant viruses, often with extensive coverage of entire viral genomes (49). All these will help us understand the overall ocular surface microbiota and provide guidance for the diagnosis and treatment of ocular surface diseases.

### Limitations

Previous studies reported that the microbial community composition changes with age. We recruited only young individuals to reduce the effects of age on ocular surface flora. Besides, the small number of control group is also a shortage. Future research may include the recruitment of participants of different ages and recruit more individuals. Longitudinal studies of these microflora related to conjunctival epithelium are needed to verify whether these bacteria are transient or stable. In addition, dropping sterile topical anesthetics and using entoiodine to sterilize the eyelid margins may affect the distribution of bacteria on the patient’s eye surface. Future research should exclude these interfering factors to explore the true distribution of microbiota on the eye surface. Genetic analysis using MinION itself has some limitations. Due to the high sensitivity of amplicon sequencing, even a small amount of DNA contaminated during the sample preparation can lead to false-positive results. It is required to minimize the risk of DNA contamination both in collecting clinical specimens and the subsequent experimental procedures. Moreover, repeated specimens from one patient are necessary to be sent for testing in some situations (50). But even if the same sample is taken from the same patient, the repeatability of the results will be poor due to multiple factors including material collection time, operator, drug use.

## Conclusion

In conclusion, with the use of metagenomic nanopore sequencing, we found that the bacterial imbalance in the CS and MG of patients with MGD and the bacterial community composition on the ocular surface of patients with severe MGD were significantly reduced. It provides us with a more detailed and realistic microbial composition of the ocular surface. These findings contribute to understanding the role of ocular surface bacterial populations in MGD and provide some basis for the indications of antibiotic or topical probiotics in future treatment directions.

## Data availability statement

The datasets presented in this study can be found in online repositories. The names of the repository/repositories and accession number(s) can be found below: NCBI BioProject, ID: PRJNA890545.

## Ethics statement

The studies involving human participants were reviewed and approved by the Declaration of Helsinki and was approved by the Ethics Committee of the Peking University Third Hospital (S2021222). The patients/participants provided their written informed consent to participate in this study.

## Author contributions

DJ: research design, data acquisition, data analysis, and manuscript preparation. XJ: research design and manuscript modification. XR: data acquisition and data analysis. JS: data acquisition. RH: data analysis. XL: research design. All authors meet the International Committee of Medical Journal Editors (ICMJE) criteria for authorship for this article and take responsibility for the integrity of the work as a whole.

## Footnotes

1
https://www.ncbi.nlm.nih.gov/biosample
